# The Effectiveness and Clinical Usability of a Handheld Information Appliance

**DOI:** 10.1155/2012/307258

**Published:** 2012-04-02

**Authors:** Patricia A. Abbott

**Affiliations:** Health Systems & Outcomes Department, Johns Hopkins University School of Nursing, Baltimore, MD, USA

## Abstract

Clinical environments are complex, stressful, and safety critical—heightening the demand for technological solutions that will help clinicians manage health information efficiently and safely. The industry has responded by creating numerous, increasingly compact and powerful health IT devices that fit in a pocket, hook to a belt, attach to eyeglasses, or wheel around on a cart. Untethering a provider from a physical “place” with compact, mobile technology while delivering the right information at the right time and at the right location are generally welcomed in clinical environments. These developments however, must be looked at ecumenically. The cognitive load of clinicians who are occupied with managing or operating several different devices during the process of a patient encounter is increased, and we know from decades of research that cognitive overload frequently leads to error. “Technology crowding,” enhanced by the plethora of mobile health IT, can actually become an additional millstone for busy clinicians. This study was designed to gain a deeper understanding of clinicians' interactions with a mobile clinical computing appliance (Motion Computing C5) designed to consolidate numerous technological functions into an all-in-one device. Features of usability and comparisons to current methods of documentation and task performance were undertaken and results are described.

## 1. Introduction

Physicians and nurses are highly mobile workers who operate in complex, stressful, and safety critical environments. Frequent interruptions, rapidly changing patient status, complex clinical presentations and information from multiple streams all combine to increase the cognitive load of practitioners and create the potential for medical error. These challenges have created a demand for technological solutions that will help clinicians manage information and make optimal decisions in this demanding work environment. The plethora and diversity of highly portable, increasingly compact, and powerful information and communication technology (ICT) devices on the market is evidence of an industry response to this growing demand.

Untethering a provider from a physical “place” with mobile technology and delivering the right information at the right time and at the right location are expectations for effective and safe clinical practice. These technological solutions can, however, contribute to the problem. Clinicians are confronted with numerous different devices to complete a series of related, yet separate actions. It is not uncommon to see practitioners with a mix of communication devices, barcode readers, and computers on wheels—some being worn around the neck, hooked to belt loops, and stuffed in pockets, while others are being pushed up and down hallways. This is in addition to stethoscopes, otoscopes, and other clinical devices traditionally carried by a provider.

This problem of device overload or “technology crowding” is now becoming an additional clinical millstone. Indeed, recent studies are pointing to marked productivity losses in environments where high technology dependence and technology overload intersect [1]. Orchestrating numerous devices with a variety of functions (some which overlap), increases clutter and cognitive load, distracting the user's attention away from the tasks at hand. Losing focus in the clinical environment contributes to increased opportunity for medical error [2, 3].

In recognition of the problem of technology crowding, a shift from numerous independent single-function devices to consolidated mobile information appliances (such as i-pads, multifunction smart phones, and portable clinical tablet PCs), is occurring. While this shift is appropriate and welcomed by most, it is dangerous to consider device consolidation as a panacea to the information management challenges raised earlier. As with any new technology, it is important to fully understand how the technology is utilized in the real-world environment, the degree of usability that it possesses, the impact it may have on users, and its effect on workflows. This is of great importance, particularly in safety-critical environments where prediction of sequelae is difficult and electronic propagation of error can be immediate and far reaching.

Studies that compare how health IT is *actually used*, versus how the device was *designed to be used,* are necessary. There are numerous instances of a misalignment of design and actual real-world use of health IT in the literature. Han et al. [4] demonstrated unexpected increases in mortality in a pediatric ICU after the implementation of a commercially available computerized provider order entry system (CPOE), while Koppel et al. [5] uncovered 22 types of medical error risks facilitated by CPOE. Ash et al. [6] specifically focused on the unintended consequences of health IT, describing how and why errors occur when health IT is implemented without investigations of how patient care systems are actually used in the real-world clinical environment. Vincente [7] makes the important point that the biggest threats to both safety and effectiveness arise from situations that are “unfamiliar to workers and that have not been anticipated by designers” (page 22).

Studies and experience show that busy clinicians will not tolerate technology, software, or processes that impose workflow barriers or that introduce additional difficulty into already complex task performance. Workarounds, a common response to suboptimal technology, are a frequent result of problems with technology design. Workarounds can result in use of the system in ways not anticipated by the designer; echoing the point made by Vincente [7]. When workarounds occur, built-in safety features are often circumvented, and the potential for a cascade of negative downstream effects can occur [8]. For example, Koppel et al. [9] cite observations of nurses who carry extra copies of barcoded patient wristbands to avoid multiple trips to the drug carts. In effect, this workaround disabled device safety alert features that resulted in wrong patient-wrong drug errors.

Workarounds and unanticipated uses of technology are becoming increasingly dangerous in healthcare environments. In this era of healthcare reform, accountability and reimbursement for “meaningful use” of health information technology, the impetus for comparisons of design intention with actual use is highly important. Improved design and reduction of the negative unintended consequences are the goals of health information technology usability and impact studies.

## 2. Study Goals and Questions

With these factors in mind, we undertook a study to gain a deeper understanding of clinicians' interactions with a mobile clinical computing appliance designed to consolidate numerous technological functions. Features of usability and comparisons to current methods of documentation and task performance while using a portable PC (mobile clinical computing appliance) were of particular interest.

The following specific questions were the foci of the study.

1. What specific themes define the usability challenges that clinicians encounter when using a mobile device to assist them in completing typical clinical tasks?
2. How usable is the C5, viewed as an important instance of a class of devices that are increasingly used by clinicians in patient care settings?

While this study focuses on one device, and the results are not generalizable beyond the specific device tested, the usability themes that emerged from pursuit of question 1 and methods employed in this study can be applied to a wide range of devices and can help guide the way usability of such devices is assessed in the future. The approach employed in this study is intended to be of particular applicability to multifunction devices such as the C5.

## 3. Methods

### 3.1. Device

We studied a newly introduced “all-in-one” mobile hand-held PC, the “Mobile Clinical Assistant” (or MCA C5 ) that was specifically developed to address the challenges of technology crowding and device overload in busy healthcare environments. The C5 mobile PC incorporates wireless technology, Windows operating system, a 10.4 inch color display screen, a barcode scanner, a digital camera, a RFID reader, and a biometric fingerprint reader. The device weighs 3.3 pounds and also has built-in loudspeakers, a microphone, a handle, and a tethered writing stylus. The C5 has a water resistant, sealed case to allow disinfection using equipment grade liquids (such as Viraguard) between patient encounters. The device is “ruggedized” to withstand a drop from 5 feet onto concrete. The C5 can access and display clinical information from external servers; no personal health information is persistently stored on the device itself. Finally, the device contains an accelerometer which enables the screen display to rotate based on device orientation, and an antitheft system which can be set to alarm, shut down, and delete all content in temporary storage if the device is moved outside the work environment, where its use is authorized.

### 3.2. Subjects

Study subjects were a convenient sample of experienced clinical nurses, recruited via word-of-mouth and by advertisement on several nursing listservs.

### 3.3. Setting

Data were collected in a simulated clinical environment as these subjects completed a series of tasks designed to reveal the strengths and weaknesses of the C5's design. We conducted both phases of this study within a large University School of Nursing 30-bed patient care simulation laboratory, and specifically in a small side classroom that is structured to represent a 3-bed intensive care unit. Within this room, there are 2 full-size Laerdal “SimMan” clinical mannequin simulators and one infant “SimBaby” in a bassinet.

### 3.4. Tasks

With simulated patient data provided by an electronic health record system (Eclipsys Sunrise Clinical Manager—SCM Version 4.5), subjects performed tasks related to barcode medication administration, digital photography of a stage 4 pressure ulcer for wound documentation, and an assessment of a newborn with documentation. Each of these tasks was chosen as representative of actions that a nurse might undertake in the course of a normal clinical workday.

For the purpose of the use of the C5 digital camera testing/wound assessment, a partial body mannequin with a variety of skin ailments was used. This partial mannequin is designed to illustrate a variety of skin conditions for use by educators. For example, a very life-like stage 4 sacral deep pressure ulcer with exposed bone, tissue tunneling, wound edges, exposed muscle, and exudate is present as sutures, rashes, stage 1 and 2 pressure ulcers, bruises, and nevi. The stage 4 sacral pressure ulcer was used for a portion of digital photography component of the study. The subjects also used a full-size SimMan mannequin to approximate camera use with a “live” patient who required turning and positioning to obtain a picture of the sacral pressure ulcer.

The barcode scanning component of the study was implemented via the use of proprietary forms software and barcodes constructed specifically for this study. Barcoded badges, medications, and patient ID bands were created and used in the testing of the C5 barcode scanner. ID bands were attached to mannequins and contrived “staff badges” with a barcode on the back were created and worn by subjects. “SimBaby” was used for the assessment procedure using the C5. All studies were completed in the same room under similar light conditions (mid-day).

### 3.5. Study Design

Following IRB review and approval, the study was conducted with two separate phases using two different subject samples. Phase 1 tested the procedure and the tooling prior to enrolling and studying the primary participants. Two experts were used for Phase 1. In Phase 1, user and environmental analyses were conducted to profile the characteristics of system users and the environment in which they interact. Heuristic evaluations and cognitive walkthroughs, a type of usability inspection where evaluators interact with the system and examine the device for usability issues, were also performed in Phase 1. This trial phase enabled the formal study procedures to be fine-tuned and the data collection procedures to be refined. The results from first part of the study will not be covered in detail in this paper.

Phase 2 of the study was conducted with 15 subjects to generate data illuminating the usability of the C5. Data were generated through ethnographic observations, surveys, and interviews of users during and after the performance of a series of the three tasks (documenting, photographing, and barcode scanning) while using the C5. The focus of this paper is on Phase 2. In Phase 2, subjects completed in random order three simulated tasks using the C5 device wound documentation using digital photography; barcode scanning with medication administration, and completion of a standard admission assessment on a newborn infant. Each participant completed the questionnaire after finishing all three tasks. Trained observers documented field observations, and subjects were asked to “think-aloud” as they worked through the scenarios.

### 3.6. Data Collection Methods and Instruments

As each subject completed the three tasks, the PI was taking notes, inquiring, encouraging think-aloud, answering, and probing/interviewing about specific actions. The field notes from the observations were included in the data analysis. The “think-aloud” protocols generated by participants were recorded directly by the C5 device and saved.

The questionnaire used in this study was adapted from the QUIS (Questionnaire for Use Interaction Satisfaction). QUIS is a long-standing, reliable, and valid usability checklist (http://lap.umd.edu/quis/). The QUIS was modified based on focus group input, adding specific items unique to the characteristics of the C5, and then content validity was determined by an expert panel in Phase 1. The resulting questionnaire was comprised of 7 sections: demographics (11 items, including years in practice and computing experience); overall user reaction (5 items); physical characteristics of device (13 items); device reliability (1 item); simulated device management activities (2 items); other topics (6 items); user opinions (6 items). Items used Likert-type response scales (e.g., Easy-Hard) or checklists (Yes-No). Each of the 7 sections also included an area for free text comments comparing the C5 with standard methods of similar task completion/documentation in clinical practice. The entire questionnaire took approximately 15 minutes to complete.

### 3.7. Study Procedure

Following consent, each subject's experience began with orientation to the C5. Subjects were taught how to use the C5 camera, the C5 barcode scanner, and how to document in Eclipsys SCM. Each subject was also oriented to the device, how to adjust the views based on arm positioning, how to use the writing stylus, how to insert and remove the device from a docking station, and how to change the battery and conduct the disinfecting procedure. Subjects were also instructed on the talk-aloud data collection procedure and asked to practice and demonstrate it prior to the start of the study to assure understanding and comfort.

The consenting and orientation took, on average, approximately 1 hour per subject. Subjects were allowed to question, practice, and repeat as many times as they felt necessary to come to a level of comfort with the device and the procedure prior to starting the study. Subjects personally determined how to hold the device and were encouraged to change positioning as necessary during the study. At that point, the study was begun, and the audio recorder (built in to the C5) was turned on. These audio files were later transcribed and analyzed. Following the completion of the study, the recorder was turned off, and subjects were given the questionnaire to complete.

### 3.8. Data Analysis and Usability Theme Identification

The PI, the research assistant, and two informatics experts assembled to code, analyze, and interpret the observational data and the subject voice recording (think-aloud) transcripts. To create the coding scheme for the transcripts, we employed an approach similar to that of Kushniruk et al. [10]. By reading three randomly chosen transcripts, all members of the team created individual lists of subject-expressed usability categories. Using a consensus process, the team then arrived at a single consolidated list of usability categories which were then used to classify and tag expressed comments in the audio files from all 15 subjects.

Each of the 15 transcripts was independently coded by two members of the team using the previously derived usability categories. Usability issues which arose and not represented in the original coding scheme were flagged for later consideration. Coding disagreements were settled by a third independent team member. The occurrence of each coded utterance was marked with a timing point so that, during analysis, the PI could return to that exact time marker on the audio file to listen and record any specific comments. The results from the coding of the transcripts were then matched to the 7 sections of the questionnaire and (along with observations from field notes) were used to complete the dataset for analysis.

The following example illustrates how the three data streams (questionnaire, observations, and coded transcripts) were consolidated. One question on the survey asked “How easy is it to use the camera during the process of documenting with the C5?” The subject's rating from the questionnaire was then supplemented with any instances from the subject's coded transcript of expressed difficulty with the camera. The PI's field notes were examined and any observations that highlighted user difficulty with using the camera were noted and added to the dataset. In example, observed difficulties with the camera included subjects struggling to depress the shutter button with the occasional accidental machine shutdown caused by hitting the on/off button located adjacent to the shutter button. The clustering of these three data streams created a deeper and multidimensional dataset of usability issues.

## 4. Results

### 4.1. Demographics

Of the 15 RN subjects, there were 2 males and 13 females. Twelve of the subjects identified themselves as White not Latino, 1 identified as Asian not Latino, and 2 identified themselves as White Latinos. All subjects were RNs; three were prepared at the baccalaureate level, ten had a master's degree, one had a PhD, and one had obtained postdoctoral training. Most of the subjects in the study were between 41–55 years of age. The average number of years of RN licensure in this sample was 21. The degree of comfort with the use of computers in the clinical setting for patient care purposes was assessed by participants as high—with all but two ranking themselves as “very comfortable.” Two ranked themselves as “somewhat comfortable.” The majority of the users estimated that they used computers in their clinical practice upwards of 50% of the time.

### 4.2. Usability Themes

The data from the questionnaire, observations, and audio recordings clustered into 5 themes. Several themes (1 and 3) included subthemes:

1. input ease (with subthemes of TIP tool, barcode reader, and camera);
2. portability;
3. security/safety (with bacterial transmission included as a key aspect of safety);
4. efficiency gains;
5. general ease/intuitiveness.

### 4.3. Usability of the C5

#### 4.3.1. Theme 1: Input Ease

The theme of “input ease” is a compilation of specific items in the consolidated data set that relate to ease by which data can be input into the C5. The input ease theme broke out naturally into subthemes based on the three different input modalities: TIP tool, barcode reader, and camera. The TIP tool was useable in two ways—by tapping and clicking with pulldown menus and onscreen keyboard or using the stylus like a pen with handwriting recognition. The TIP tool is not specific to the C5, it is a Microsoft feature, yet many of the subjects had no experience with the use of a TIP tool. It is included here due to its relative negative impact on usability comparisons.

**TIP Tool**

The results of the use of the TIP tool stylus-based input met with mixed results. Eight of the 15 subjects rated the TIP tool “tapping” input as somewhat to very difficult, and the field notes and coded comments revealed marked instances of difficulty and frustration. Subjects were observed to repeatedly tap the screen with increasing vigor while and expressing negative perceptions. In contrast, the TIP tool handwriting recognition was rated positively by 13 of the 15 subjects, with many expressing surprise at its level of accuracy. However, only 1 of the 15 subjects mastered the proper method of editing the handwriting, spawning creative yet inefficient workarounds. Frustration with the editing function was high, but the perceived value of being able to handwrite on the screen was a highly rated feature amongst most of the subjects.

**Camera**

Eighty percent of the subjects rated the digital camera built in to the C5 as a very positive feature of the C5. The participants voiced support for digital photography as a part of the patient record and believed that the impact of the camera on workflow and patient care was overwhelmingly positive. Recorded comments relayed comparisons with current methods of photography in clinical settings which revealed very inefficient processes of requesting a camera, locating it, assuring that the batteries were operational and similar. Several subjects stated that they would enjoy using such a camera when working with patients in chronic wound management settings to show the status of wounds that a patient could not easily visualize (such as sacral pressure ulcers) or to better document the nature of wounds for a patient record. While supportive of the camera as a concept, 11 of the 15 participants found the C5 camera difficult to use. Problems included the location of the shutter button adjacent to the on/off switch, the positioning of the stylus tether directly in front of the lens, the low megapixels (2.0) which resulted in lower quality photos, and poor flash strength. In addition, subjects did not respond favorably to the process of focusing which required that the entire C5 be moved in and out (similar to an i-Pad) instead of being able to autofocus or zoom in with a focus button on the device itself.

**Barcode Scanner**

Usability of the barcode scanner was rated highly, with only 2 of the subjects rating the scanner to be “somewhat difficult” to use in the survey. The observational and the coded transcript data, however, provide additional dimensionality to the use of the barcode scanner and opportunities for improvement. In analysis of the remarks, the subjects were overwhelmingly positive about barcode scanning and were pleased that the C5 contained this feature. However, subjects voiced a concern about having to move the entire device to scan something, and about the limited range of the scanner (6–8 inches maximum). For example, the testing scenario included scanning an IV bag that was already hanging from a pole. One subject reached over the mannequin to scan a barcoded IV bag and dropped the device on the mannequin's head. Several expressed concerns about ease of scanning a patient's wristband and having to position the entire C5 device to do so.

Six subjects verbalized the value of bar coding and viewed it as an important safety feature. Others commented that it was good to have an “all in one device” because they were “already loaded with things to carry” and were not in favor of a documentation device and a separate barcode scanning device. Three subjects who were familiar with barcode scanning also commented that a barcode scanner located away from where scanning occurs “does not help me to improve safety or make my job easier” (paraphrased).

#### 4.3.2. Theme 2: Portability

The portability theme included the benefit of being “untethered” from a fixed workstation in addition to perceptions of transportability/handling of the device. The portability of the device was rated from “valuable” to “very valuable” by 11 of the 15 participants on the survey. The transcripts and observation data supported the survey results with many verbalized comparisons of current practice with fixed workstations and the inefficiency of computers on wheels and/or fixed stations.

At the start of the study, every subject was encouraged to hold and readjust the C5 as needed and to use the built-in handle as he/she saw fit. Observational and transcript files reveal significant amounts of shifting and repositioning of the device that decreased over time. The autorotation of the screen was voiced by several participants as a necessary and positive feature. Five of the 15 participants asked for an accompanying “strap” of some sort so that they could have two free hands at times. Three other participants said that a strap would alleviate some of the concerns they had about the device weight. Twelve of the 15 subjects carried the device like a lunchbox in between task stations in the lab. Most of the subjects were observed to use the device like a clipboard or a medication tray.

While the majority (60%) of the participants rated the device's weight (3.3 lbs) on the survey as “neutral”, all other ratings were skewed towards intolerable. The observational and transcript data highlighted concerns over weight, yet at the same time illustrated resourcefulness of the nurse subjects to adjust. Eight subjects specifically commented on the weight as being a problem, yet 5 of the 8 simply determined a way to deal with it (e.g., pulling up a bedside table, putting it on the edge of the bassinette, balancing it on a side rail or bedside table, or propping it on their knee). This also spawned the request for a strap or somewhere to hang the device when hands were needed for something else.

#### 4.3.3. Theme 3: Security and Safety

The theme of “security/safety” is a compilation of specific items in the consolidated data set that relate to the perceptions of security and safety aspects of the C5 device. The concept of ability to disinfect the C5 was included in this construct as a patient safety dimension.

Participants rated the ability to disinfect the C5 as a “very important” feature (**N** = 13) and as making an important contribution to ease of use and efficiency. Regarding theft and data security, six of 15 leaned more towards “very worried,” while 7 were on the opposite end of “not very worried.” The survey results also revealed that most of the subjects were not concerned about the security of patient data on the C5, with thirteen of the 15 subjects having “little to no concerns.” In the transcripts, two subjects voiced concerns that patient data “lives” on the C5 even after being explained that the C5 is just a conduit to the server. These two subjects were adamant, fearing that if the device was stolen someone could access a copy of patient data that resides inside of the C5. Six of the subjects expressed concern that the C5 would be appealing to thieves and also that the clinicians would be held responsible if the device were stolen. 

#### 4.3.4. Theme 4: Efficiency Gains

The theme of “efficiency gains” is a compilation of variables from the consolidated data set that relate to the potential contributions that the C5 device may make to efficiency and usefulness. The process of wipe disinfecting the device clustered with this construct due to comments about time savings and/or additional steps that may facilitate efficiency in workflow.

The overall usefulness of the device was rated highly positive on the survey, with 13 subjects indicating that the C5 would help improve their practice. The transcripts and observational data support the survey data. Comments included “No more running back and forth, forgetting and missing details. I have the machine where I need it and when I need it” and “In the morning, we have so many services on the floor, everyone is looking up their labs, and all the computers are taken up and nurses cannot get to their POE orders because they cannot get to the computer. This will allow them to have their own POE orders in their hands, and not have to worry about fighting a resident for a computer system first thing in the morning.”

Similarly, 13 of the 15 subjects on survey believed that the C5 will improve their efficiency and effectiveness. The transcript and observational data support the survey data. Comments included “The disadvantage (of) coming out to the station is that you always get interrupted and then you (find that you) forgot to document, whatever. So the faster you can document, related to the actual care is better. So I think the closer to care is good” and “not walking back and forth to the nurse's station saves me time and steps. I do not have the enough energy or the memory to waste anymore.”

#### 4.3.5. Theme 5: General Ease/Intuitiveness

The theme of “general ease/intuitiveness” is derived from the variables that relate to the overall ease of using the device and the ability to “figure out” how to do something with the C5 relying on intuition and experience.

On the survey question of “overall impression of the C5 device,” the majority of the participants rated the C5 device highly. Eleven subjects rated the C5 as a “4” (approaching “wonderful”), and “4” ranked it with a “5” (wonderful). On the survey scale that assessed frustration versus satisfaction—8 of the subjects felt that the device was frustrating (8 ranked it as neutral or worse) to use. Similarly, 7 of the 15 rated the device as somewhat difficult to use. However, ten of the fifteen ranked the use of the device as stimulating or very stimulating (in contrast to boring or dull) to use. Most of the subjects (9) rated the C5 as “intuitive and easy to use.”

The results of the observation data shed additional light on the seemingly contradictory findings from the survey. Those who had an observed higher level of computer experience appeared to be more “at ease” with the device and used the features much more easily. This observation may illustrate differences between self-rated levels of computing experience (which were high by survey) with actual ability. For example, even though the majority of survey results pointed towards high level of comfort and computing literacy, subjects who were familiar with the TIP tool were observed to readily use it without issue. Those subjects who were very familiar with Eclipsys SCM 4.5 software had apparent/observed higher levels of comfort. Subjects with a greater degree of computing experience were able to open and close applications easier, use the barcode scanner, increase sizes of windows to enhance visibility, and readjust the view (portrait/landscape) to adapt to needs. Others struggled with certain aspects of the device and their frustration was apparent to the observers. Examples of comments from the transcripts were “Do something with the string, it is driving me crazy”; “I can do this quicker with a pen and paper, the handwriting recognition is not working for me”; “How do you minimize something…actually, what does minimize mean?”

## 5. Discussion

On the whole, the study participants perceived the C5 as highly useful, believed that the device would contribute to efficiency gains in practice, and considered device portability to be very important in supporting clinical workflow. The subjects' comparisons of the C5 with standard and current personal practice revealed significant frustration with the redundancy of current methods of documentation, device overload, and the imperative of employing workarounds when inefficient processes impede timely completion of tasks in busy environments.

The ability to quickly disinfect the device and move on to the next patient was clearly important to the nurses who were the subjects in the study, particularly in consideration of an increased focus on prevention of hospital acquired infections. Compared with current methods for documentation and performance of the tasks the C5 supports, the subjects valued the ability to untether from the nurse's station and be able to access and enter data instantaneously at the point of need. In addition, the value of having a personalized portable computing device and not having to compete for a workstation, particularly during shift change or rounds, was a virtue of the C5 raised by subjects. Barcoded functions are increasing in popularity, and the subjects expressed strong desire for not being loaded with another device or having to pull a computer on wheels with an attached barcode scanner into the room. Smaller, more portable, and all in one appeared to be the most desirable mechanism for this study population.

The untethering potential of the C5 may have implications beyond ubiquitous access to data. Empowered by a portable multifunction device, clinicians began to imagine novel ways the technology could be used to help them in their daily work. Several of the subjects who specialize in ostomy and wound care began to generate ideas about exchanging wound pictures across the team to measure healing responses, to be able to take a picture of a sacral ulcer to show a patient the impact of a certain treatment or the benefits of an action the patient and or family has taken, or to take a picture of a patient as part of the formal medical record so that proper patient identification at bedside is enhanced. Digital photography incorporated as part of wound care assessments was viewed by several of the participants as a more accurate method of documentation than the current practice of narrative description.

Even in light of the overall positive reaction to the concept of an all-in-one portable computing device, distinct usability issues emerged from the study. Some of the identified usability issues were potentially serious and could have negative consequences, from user frustration and possible technology abandonment, to patient harm. The study revealed many aspects of the device that could be improved with design modification and also perhaps through enhancing training and increasing computer literacy in clinical user groups [11]. The aspects of the device most in need of attention, in the view of study subjects, were centered on “form factor” or physical device form. The areas of improvement in regards to the form factor included:

1. the location of on/off switches next to other important feature buttons. Frustration was high when, after arranging the patient and the device to take a picture, the off switch was accidentally pressed instead of the shutter and the machine shut down. It took considerable time to restart and reauthenticate, reposition the patient and refocus, generating negative subject reactions;
2. the location of the stylus tether which results in its hanging over the camera lens. After taking a sometimes difficult to obtain picture, users were quite frustrated with the appearance of the tether;
3. the weight of the device without some way to offload it easily to reduce weight stress and/or free up hands. As the study procedure time progressed, subjects began to voice concerns about the weight and what 8 or more hours of use would invoke;
4. the camera structure with no auto focus or ability to adjust lens without moving the device and the low megapixels of the camera. The manner of focusing (similar to that of an i-Pad) was not positively received, and the low resolution thwarted some of the benefit of wound documentation where edges and color resolution are very important aspects;
5. the need for detachable/retractable components to better support workflow, such as the camera and the barcode scanner on a tether to support higher maneuverability around a patient. Subjects suggested that a camera lens or the barcode reader be put in the stylus (or similar) so that they could stretch it to the patient instead of requiring the movement of the entire device to the patient.

Other areas of improvement were noted that are not related to the physical form factor, and fell instead on aspects related to the subjects themselves. Approximately half of the subjects had concerns about the security of patient data on a portable device, a view that persisted after discussions of how client-server technology eliminates persistent data storage on the C5. The subjects' belief about data persistence was difficult to change. An additional aspect was in the observed difference between self-reported computer comfortableness/literacy and the observed levels of the same. Even though the demographics in the survey illustrated that all but 2 of the subjects felt “very comfortable” with computing technology and that over 50% said that they routinely use computing technology in the workplace, there were observable differences in comfort and agility of use of the device. Nurses who were observed to be more comfortable with computing technology had lower levels of frustration, and more easily configured the device to fit their style. Several subjects struggled with basic computing manipulations such as minimization, how to work with pull down menus, and moving between landscape, and portrait orientations. The findings point to a need to enhance the general computing competencies of all clinicians—who are expected to be able to work with increasingly complex health IT.

An additional potentially valuable outcome of this study in a specific example of health IT usability is in the five themes that emerged from the multimethod approach. With the expectation that more devices of this type will come on the market with similar design characteristics, a structure for quickly assessing the general dimensions of usability may be a useful tool. Further study and validation is needed, however, particularly in naturalistic settings where additional external influences will further impact use patterns and potential workarounds.

The primary limitation of the study is the focus on a single device with multiple features that have been encapsulated in a specific form factor. As such, the results speak to the usability of this single device in toto. While many of the findings may carry forth to support general usability principles (e.g., the suboptimal placement of the on and off button adjacent to the shutter button), this study was not able to measure the contributions of individual features to overall measures of usability.

Finally, generalizability of the usability themes that emerged from this work must necessarily be the subject of further research. These themes may prove to be limited to multifunction devices such as the C5 or they may generalize more widely. Further research that focuses upon consolidated devices such as the C5 and their impact on usability is warranted.

In general, the study resulted in overall positive findings regarding the utility and usability of a portable information appliance, particularly in comparison to current methods used by the participants in similar clinical situations. The usability constraints that arose were primarily related to the physical form factor, issues that can be mitigated with further design modification. The need for mobile and highly usable devices to support the effectiveness of busy clinicians is high, and further studies of the alignment between design intention and real-world use are imperative.
